# Team Resilience as a Second-Order Emergent State: A Theoretical Model and Research Directions

**DOI:** 10.3389/fpsyg.2017.01360

**Published:** 2017-08-17

**Authors:** Clint Bowers, Christine Kreutzer, Janis Cannon-Bowers, Jerry Lamb

**Affiliations:** ^1^Department of Psychology, University of Central Florida Orlando, FL, United States; ^2^Cannon-Bowers Consulting Orlando, FL, United States; ^3^Naval Submarine Medical Research Laboratory Groton, CT, United States

**Keywords:** team performance, resilience (psychology), stress, psychological, team processes, team training

## Abstract

Resilience has been recognized as an important phenomenon for understanding how individuals overcome difficult situations. However, it is not only individuals who face difficulties; it is not uncommon for teams to experience adversity. When they do, they must be able to overcome these challenges without performance decrements.This manuscript represents a theoretical model that might be helpful in conceptualizing this important construct. Specifically, it describes team resilience as a second-order emergent state. We also include research propositions that follow from the model.

## Introduction

In 1914, Sir Ernest Shackleton and his team set out from Plymouth, England on a quest to walk across the Antarctic continent. The goal was to be the first person to successfully cross the 1,500 miles of frozen tundra. Upon stopping at a whaling station as they set out on their quest, the team found itself stuck on ice. They spent nearly 11 months by the ice-bound ship, until the ice crushed it, eventually causing it to sink. After spending a week rowing in lifeboats, the team arrived at Elephant Island. The small island offered no protection or resources. Shackelton strategized, and devised a small subset of his team members to travel 800 miles back to the whaling station they previously left in order to seek help. After rowing for 17 days, they arrived, only to realize they were on the wrong side of the island. Approximately 22 miles of ice and mountains stood them and the whaling station. However, they managed to make it to their destination in 36 h. Given the ice and storms, it took Shackleton nearly 3 months to rescue the remaining men on Elephant Island. More than 2 years after leaving Plymouth port, all of the men had finally returned. The story of Shackleton story is so compelling due to the resilience he and his team members displayed. While it took more than 2 years, everyone returned home safely due to the resilience displayed by Shackleton and his team.

## Why is team resilience important?

Resilience has been recognized as an important phenomenon for understanding how individuals overcome difficult situations (Masten and Osofsky, 2010). However, it is not only individuals who face difficulties; it is not uncommon for teams to experience adversity. When they do, they must be able to overcome these challenges without performance decrements. Indeed, research has identified a multitude of stressors that teams often face, including: poor interaction quality, poor communication channels, lack of back-up behavior, and negative organizational culture. While these stressors have been identified, the exploration of how teams can utilize their collective resources to overcome them has been largely overlooked. However, focus on resilience has recently grown, as researchers attempt to identify how teams and groups positively adapt to adversity (West et al., 2009; Bennett et al., 2010; Morgan et al., 2013, 2015; Alliger et al., 2015). It appears as though team resilience is a critical team level capacity that facilitates the rebound of teams after an adverse event. In light of this definition of resilience as a capacity, resilience can be seen as a buildable capacity. Teams that thrive, rebound, or positively adapt to adversity are more unlikely to experience the deleterious effects of challenging situations.

## Definitions of resilience

The term *resilience* comes from the Latin word “resiliere,” which means to “bounce back”; it typically refers to the ability to recover or rebound after a setback (Fletcher and Sarkar, 2012). Indeed, the concept of resilience has been deemed an important phenomenon for understanding how successful adaptation occurs following an unanticipated—often negative—event (Wright and Masten, 2015). Interest in studying resilience as a coping or adaptation mechanism has increased rapidly over the last 20 years, and is considered across a variety of contexts, such as communities (Brennan, 2008), teams (Pollock et al., 2003), education (Gu and Day, 2007), organizations (Riolli and Savicki, 2003), military (Palmer, 2008), and athletic performance (Galli and Vealey, 2008).

As interest in resilience rises, a number of definitions and conceptualizations have been put forth in the literature. Not surprisingly, one of the primary shortcomings of previous resilience research is the wide discrepancy regarding its definition and conceptualization (Fletcher and Sarkar, 2012). More specifically, resilience has been referred to sometimes as a trait, other times as a process, and yet other times as an outcome. Davydov et al. (2010) assert that these discrepancies and definitional confusion have hindered the evaluation and validity of resilience research findings. To complicate matters further, resilience has been studied at different levels of analysis. Traditionally, resilience has been used to refer to individuals, but more recently has been applied to teams and organizations. The sections that follow define resilience as it has been used at these various levels.

## Resilience as a process

Resilience researchers have shifted to examining resilience as a dynamic process, rather than an enduring trait. As a fluid process, some have proposed that resilience gradually develops over time, through interactions between the individual and the environment (Egeland et al., 1993). Most scholars agree that within the process, there is a complex interaction of multiple factors that determines whether resilience is demonstrated.

In line with the notion of resilience as a process, Galli and Vealey (2008) found that a significant facet is *agitation*, a process in which unpleasant emotions or mental struggles are countered through various coping strategies. Notably, positive adaptation occurs gradually, and requires frequent shifts of thought. These findings can be nested within the context of contemporary stress and emotion theory, which suggests that individuals construe relational meanings based on their interactions in a given environment (Lazarus, 1998). Similarly, a recent theoretical model offering insight into resilience is the “Meta-Model of Stress, Emotions, and Performance” (Richardson, 2002). The model suggests that suggests that stressors are created in the environment, become mediated by perception, appraisal, attribution, and coping, and finally, result in adaptive or maladaptive stress responses. The relationship between these processes and responses are further moderated by situational and individual level characteristics, including self-esteem, positive affect, and self-efficacy (Schaubroeck et al., 1992; Ganster and Schaubroeck, 1995; Schaubroeck and Merritt, 1997). These characteristics affect stress processes at several points, including stressor appraisal, meta-cognition in response to affect, and coping strategy selection.

Other researchers have also emphasized the role of stressors in the development of team resilience. For example, Meneghel et al. (2016) emphasize the role of job demands in the development of team resilience. However, their data indicate that there is a more complex relationship between job demands, resources, resilience, and performance than one might expect. More specifically, job demands may induce stress and thereby hamper positive emotions, thereby decreasing team resilience. However, when job demands do not place too much workload on team members, this may lead to a sense of accomplishment, thereby inducing positive emotion and the facilitation of resilience.

In his work, Richardson (2002) defines resilience as “the process of coping with stressors, adversity, change or opportunity in a manner that results in the identification, fortification, and enrichment of resilient qualities or protective factors” (p. 308). According to the theory, the process of resilience begins at the state of “biopsychospiritual homeostasis” (i.e., a comfort zone), in which an individual is physically, mentally, and spiritually in balance. This state is disrupted if an individual does not have sufficient protective factors to buffer strains, stresses, or adverse events. Over time, an individual will adjust and begin the process of reintegration. The reintegration process results in one of four outcomes: (1) resilient reintegration (additional protective factors are attained or strengthened, and homeostasis is once again achieved) (2) homeostatic reintegration (an individual remains in homeostasis, just “getting past” the situation), (3) reintegration with loss (protective factors are lost, and a lower level of homeostasis is achieved), or (4) dysfunctional reintegration (individuals resort to destructive behaviors) (Richardson, 2002).

Morgan and his colleagues point out that team resilience also has elements of a developmental process (Morgan et al., 2015). They conducted a narrative analysis of world-class rugby players. The results of this analysis suggest that team resilience might be developed during different phases of the team's development. For example, early development of resilience might be characterized by behaviors designed to increase collective efficacy. However, more mature teams focused on dealing with failures.

As previously noted, the notion of resilience as a process has also been well-developed at the organizational level. According to this body of work, resilient organizations treat deviations from boundary conditions indicators of overall system health. Resilient organizations behave as high reliability organizations (HROs). These organizations overcome adversity with few to no errors due to their “intelligent wariness” (Reason, 2000) and a “preoccupation with failure” (Weick and Sutcliffe, 2006). Resilient organizations intentionally test their risk assumptions and assumptions regarding overall system health (Weick and Sutcliffe, 2006). Furthermore, highly resilient organizations empower their employees to speak up to report errors or conditions that could foster errors. These organizations recognize that speaking up is critical, even if production is halted to mitigate a foreseeable potential error. Moreover, resilient organizations believe they have the capability to cope with a plethora of stressors, and continuously strive to strengthen their resources to do so. Therefore, resilient organizations acknowledge that they are imperfect, but believe they can grow by learning from near events and actual events (Woods, 2006). While this work has been conducted at the organizational (rather than team) level, given that this is the most well-developed area of resilience research, we believe this work can be translated into lessons for building team resilience. For example, given that resilient organizations encourage speaking up to report errors and are capable of handling high amounts of stress, incorporating techniques to encourage communication and cope with stress into team training may be key to facilitating resilience at the team level.

Resilience also requires practices that facilitate competence, and encourage growth to buffer against jolts and strains (Vogus and Sutcliffe, 2007). Such capabilities facilitate resilience by expanding informational inputs, creating flexibility, and reconfiguring resources. Teams have the ability to continuously grow and refine their capabilities, which in turns allow them to have greater predictive abilities, remain flexible, and buffer the detrimental effects typically associated with unexpected or negative events.

The resilience mechanisms outlined above result from and encourage a unique way of “seeing.” Organizations that are resilient are more likely to be composed of teams that are capable of elucidating weak signals through the monitoring of current operations. As such, these teams are better equipped to identify weak signals because of their highly developed response capabilities, which allow them to respond more adaptively to a great array of events. Moreover, given their superior information processing systems and management, disruptive or negative events are treated as opportunities as opposed to threats (Jackson and Dutton, 1988; Barnett and Pratt, 2000). For example, teams in HROs use “near misses” to assess the overall functioning of the system and view them as opportunities for learning (Weick and Sutcliffe, 2006).

Moreover, *teams* in resilient organization tend to engage in mindful organizing (Weick et al., 2008). This entails the ongoing development and refinement of a shared understanding of problems faced by the organization and the resources and capabilities available to maintain safe performance. Vogus and Sutcliffe (2012) suggest that mindful organizing is the result of five processes: (1) assessment of possible and extant system risks, (2) questioning of previous assumptions, (3) discussion of individual, team, and organizational resources and abilities, (4) collective learning following an adverse event, and (5) deference to expertise. When employees engage in these processes, organizations are better equipped to identify errors in a timely manner, thereby minimizing detrimental outcomes.

Conceptualizing resilience as a dynamic process allows scientists to create hypotheses about the conditions and behaviors that lead to resilience. Viewing resilience as a process may be useful, as process theories “often deal with the evolution of relationships between individuals or team members, or with the cognitions and emotions of individuals as they interpret and react to events” (Langley, 1999, p. 693). As such, process theories often involve a plethora of quantitative and qualitative information. Although this can make interpretation and analysis quite difficult and complex (Langley, 1999), taking a process view allows us to more precisely parse out the components, events, and relationships underlying resilience.

## Team resilience as an emergent state

Many team researchers have tended to focus on the construct of adaptability—in particular task adaptability (i.e., the ability to shift strategies in response to changing situational or task demands)—but these treatments may not capture the essence of resilience. Recently however, the notion that resilience is best considered an *emergent state* has been proposed (Maynard and Kennedy, 2016). The term emergent state was proposed by Marks et al. (2001) to describe certain types of team phenomena that were not actual processes (although they had been treated as such in prior work). According to Marks et al. (2001), “Emergent states describe cognitive, motivational, and affective *states* of teams, as opposed to the nature of their member interaction. Although researchers have not typically classified them as such, emergent states can be considered both team inputs and proximal outcomes. For example, teams with low cohesion (an emergent state) may be less willing to manage existing conflict (the process), which, in turn, may create additional conflict that lowers cohesion levels even further” (p. 357). The authors go on to clarify that emergent states are not actual team actions or interactions; rather, they should be viewed as an outcome of team experiences, including team processes.

Maynard and Kennedy (2016) view team resilience as an emergent state, given the idea that resilience is dynamic (Luthar et al., 2000) and is impacted by adaptation (among other team processes) (Moran and Tame, 2012). Reich et al. (2010) purport that resilience is the result of adaptation to difficulty, which is in line with the notion of team resilience as an emergent state. Similarly, conceptualizing resilience as an emergent state is in line with work that has defined it as “a team's belief that it can absorb and cope with strain, as well as a team's capacity to cope, recover and adjust positively to difficulties” (Carmeli et al., 2013, p. 149).

The manner by which various states emerge has been well-articulated in the context of team learning by Kozlowski and Bell (2008). Kozlowski and Bell (2008) suggest three central tenets of team learning. First, it is unquestionable that learning occurs within individuals. Next, while learning can occur at the individual-level, team learning occurs in a task and social context that shapes how learning occurs and what is learned. Finally, team learning is a dynamic process, occurring over repeated interactions over time, resulting in emergent outcomes suggesting that learning has taken place.

The value of the emergent state construct has been demonstrated empirically in recent team research. For example, Jehn and colleagues recently demonstrated that certain emergent states mediated the relationship between conflict and team performance (Jehn et al., 2008). Similar results were obtained by Bradley et al. (2012).

Employing Marks et al.'s (2001) definition, Maynard and Kennedy (2016) incorporated the concept of team resilience as an emergent state in a model of team adaptation. According to these authors, “the construct of resilience (at both the individual and team-level of analysis) has been viewed as a trait, a process, and as an outcome” (p. 8). They concluded, however, that team resilience is best thought of as an emergent state in the manner described by Marks et al. (2001). Team resilience as an emergent state suggests underlying dynamic properties that may shift as a result of team-level inputs, context, processes, and outcomes.

A similar position has been articulated by Sharma and Sharma (2016). These researchers sought to develop a measure of team resilience. A result of their scale development work was a model in which team resilience is a consequent of various latent variables comprised by more specific behaviors. While they did not invoke the construct of emergent states, their resulting model implies a multi-level process in the development of team resilience.

Our conclusion is similar: resilience is the result of a dynamic process that effects and is affected by other salient team variables. In fact, we argue that team resilience may be a “second-order”emergent state; that is an emergent state that is actually the result of other emergent states in the team. Indeed, team resilience may mediate the relationship between other team emergent states and outcomes during times of stress.

## Individual resilience

In regard to inputs at the individual level, there is growing research regarding how individual member qualities influence team adaptability (LePine, 2003, 2005). As an example, LePine (2005) revealed an interaction between the difficulty of a goal, and learning orientation. Teams that had difficult goals that consisted of team members with a learning orientation had higher rates of adaptation. As suggested by Maynard and Kennedy (2016) “We can envision more work at the team-level of analysis leveraging such individual-level work by either aggregating such individual-level constructs or by examining upward influence-type models (e.g., Mathieu and Taylor, 2007)” (p. 22).

Despite increased interest in resilience, there remains definitional debate regarding what exactly it means to be a resilient individual. More specifically, it is yet unclear whether resilient individuals thrive (i.e., grow beyond baseline functioning) or more simply adapt and return to baseline functioning after facing a setback. In line with the latter idea, Masten et al. (1990) define resilience as “The process of, capacity for, or outcome of adaptation despite challenging or threatening circumstances” (p. 426). Similarly, Lee and Cranford (2008) define resilience as “The capacity of individuals to cope successfully with significant change, adversity, or risk” (p. 213). However, other authors purport that resilience goes beyond adaptation to adversity. For example, Leipold and Greve (2009) define resilience as “An individual's stability or quick recovery (or even growth) under significant adverse conditions” (p. 41). Moreover, Connor and Davidson (2003) suggest that resilience is “The personal qualities that enables one to thrive in the face of adversity” (p. 76).

Despite this uncertainty, Fletcher and Sarkar (2013) pointed out that definitions of resilience are typically founded upon two fundamental notions: adversity and positive adaptation. In fact, researchers generally agree that positive adaption to adversity must be evident in order for resilience to be demonstrated. Luthar and Cicchetti (2000) asserted further that *adversity* “typically encompasses negative circumstances that are known to be statistically associated with adjustment difficulties” (p. 858). In addition, according to Davydov et al. (2010), the mechanisms underlying resilience vary, ranging from mild adversity (e.g., stress at work) to strong adversity (e.g., bereavement). Regarding the second underlying concept, positive adaptation “may be likened to a springboard that propels the survivor to a higher level of functioning than that which they held previously” (Linley and Joseph, 2004, p. 602). In line with this definition, positive adaptation therefore represents a gain following the adverse event, as opposed to recovery from the loss or homeostatic return to baseline.

Others (e.g., Luthar et al., 2000) suggest that positive adaptation simply refers to the ability to meet the demands faced during adversity. Furthermore, others assert that positive adaptation may be a combination of the previous definitions; Leipold and Greve (2009) suggest that positive adaptation refers to “An individual's stability or quick recovery (*or* even growth) under significant adverse conditions” (p. 41). Thus, the definitional debate in the resilience literature seems to surround the second core process of adaptation. Luthar and colleagues (Luthar et al., 2000; Luthar, 2006) suggest that positive adaptation may be a function of the severity of the adverse event, and what constitutes positive adaptation might be context specific.

Alongside definitional confusion, there has been considerable debate about the basic conceptualization of resilience. Although all people possess some degree of resilience, not everyone is equal in this regard. While some people have difficulty overcoming commonplace hassles, others react positively in the face of even the most challenging situations (Bonanno, 2004). In search of an explanation for this variance, early resilience researchers sought to identify factors that protect individuals from experiencing adverse effects after a setback. In this regard, resilience can be conceptualized as an amalgamation of protective factors, or traits, that “influence, modify, ameliorate, or alter a person's response to some environmental hazard that predisposes to a maladaptive outcome” (Rutter, 1985, p. 600). This conception was originally suggested by Block and Block (1980), using the term “ego resilience” to reflect traits such as resourcefulness, character, and flexibility. Those high on ego resilience were found to be energetic, optimistic, and had the ability to detach in order to problem solve (Block and Block, 1980). Since the origination of this work, there seems to be general agreement that the construct of resilience implies a protection against future stressors (Fletcher and Sarkar, 2016).

Several specific protective factors have been examined by resilience researchers, including: positive emotions (Tugade and Fredrickson, 2004), hardiness (Bonanno, 2004), self-efficacy (Gu and Day, 2007), extraversion (Campbell-Sills et al., 2006), self-esteem (Kidd and Shahar, 2008), positive affect (Zautra et al., 2005), and spirituality (Bogar and Hulse-Killacky, 2006).

## Team resilience

Given the growth of teamwork within organizations, resilience researchers have recently shifted their focus from the individual and community levels to the team level (Norris et al., 2008; Alliger et al., 2015). As recently suggested by Brodsky et al. (2011), “a focus on the individual is not enough” (p. 233). In line with Alliger et al. (2015), we purport that individual and team resilience while related, are distinct constructs. A team comprised of resilient members does not necessarily make the team resilient. At the team level, resilience has been characterized by variables including collective efficacy, creativity, cohesion, social support, and trust (Gittell et al., 2006; Norris et al., 2008; Blatt, 2009). Moreover, teams that encompass a broader perspective in the face of adversity have a greater likelihood of positive adaption (Bennett et al., 2010). In support of the notion that team resilience research is critical, Bennett et al. (2010) purports that, “resilience may be viewed as much a social factor existing in teams as an individual trait” (p. 225). This would suggest that teams have the capacity for positive adaptation through collective interactions, rather than as isolated individuals. As stated by West et al. (2009), “Team resilience may prove to be an important positive team level capacity that aids in the repair and rebound of teams when facing potentially stressful situations. Teams which display the ability to either thrive under high liability situations, improvise and adapt to significant change or stress, or simply recover from a negative experience are less likely to experience the potentially damaging effects of threatening situations” (p. 254).

## Organizational resilience

As noted by Maynard and Kennedy (2016), research is lacking on the effect of organizational-level inputs on team resilience. Work by Gibson and Birkinshaw (2004) have suggested organizational context to be a pre-cursor to team ambidexterity. More specifically, the more supportive the context, the greater the ambidexterity. Team ambidexterity “allows teams to reconcile the tensions between alignment and adaptability” (Maynard and Kennedy, 2016, p. 12). Moreover, Gibson and Birkinshaw (2004) found that ambidexterity is a mediator between context and unit performance. Thus, the contextual inputs at the organizational level seem to facilitate unit adaptation.

As defined by Vogus and Sutcliffe (2007), resilience at the organizational level refers to the ability to maintain positive adjustment to difficult situations, such that the result is a stronger and more resourceful organization. Since organizations that are resilient as a whole have greater resources, this may allow their individual teams to also be more resilient as they have access to a greater repertoire of resources when faced with a difficult situation. “Difficult situations” include crises, unexpected events, deviations from boundary conditions (i.e., deviations from normal functioning), strains, and emerging risks. It is important to note that the amalgamation of small stresses, deviations, or interruptions can pose a significant risk to system functioning just as readily as a more catastrophic event (Rudolph and Repenning, 2002). Adjustment to adversity at the organizational level has been said to strengthen individual teams through “a hierarchical integration of behavioral systems whereby earlier structures are incorporated into later structures in increasingly complex forms” (Egeland et al., 1993, p. 518). Alternatively stated, resiling from difficult conditions necessitates the activation of latent resources. Therefore, resilience encompasses more than a specific adaption. Competence in the face of one adversity implies a greater likelihood of competence in the face of the next adversity. In order to be resilient, a team must be prepared for hardship, which requires an “improvement in overall capability, i.e., a generalized capacity to investigate, to learn, and to act, without knowing in advance what one will be called to act upon” (Wildavsky, 1991, p. 70). In this light, resilience greatly depends on learning from previous experiences and adversities which facilitates future learning. However, because resilience is independent of learning activities, it represents a greater repertoire of capabilities.

Several resilience processes at the organization level have been identified by Brodsky et al. (2011), which include: a sense of community, positive team culture, reframing of stressors, striving to achieve the organization's mission, shared values, and malleable team structures (Fletcher and Wagstaff, 2009; Wagstaff et al., 2012). This supports the contention of Chan (1998), who suggested that although constructs may fall under the same domain, they manifest differently at different levels (i.e., individual or team). A similar position has been advocated more recently by Morgan et al. (2013).

## An input-mediator-outcome (IMO) model of team resilience

What follows is our attempt to synthesize past work to create a model of team resilience by employing a modified Input-Process-Outcome (I-P-O) framework advocated by Ilgen et al. (2005): the Input-Mediator-Output-Input (I-M-O-I) framework. According to Ilgen et al. traditional I-P-O models failed to account for the dynamic complexity that characterizes team behavior. Using Marks et al.'s (2001) notion of emergent state described above, they substitute the term “mediator” for “process” in the original I-P-O framework. In doing so, these authors contend that it “reflects the broader range of variables that are important mediational influences with explanatory power for explaining variability in team performance and viability,” (Ilgen et al., 2005, p. 520). The following sections first summarize past work into the inputs, processes and mediators, and outcomes associated with resilience as the individual, team, and organizational levels. We included the individual levels in our review because they are part of the dynamic system that effects team resilience. Our contention is that it is essential to maintain this multi-level view in order to understand the full complexity of team performance and outcomes. Based on this review, we conclude by offering a comprehensive model of those things that contribute to development of resilience and outcomes that can be expected as a result of achieving resilience. We hope this model will stimulate further thinking and research.

## Beginning with the end: defining outcomes of resilience

To begin specification of an I-M-O model of resilience, we reviewed literature summarizing the outcomes that are expected to result from resilient behavior. Our goal here is to synthesize what has been theorized about the expected outcomes of resilience at the individual, team and organizational levels (see Table 1). Implicit in all of these outcomes is that they must occur during a period of stress that is sufficient to interrupt performance.

**Table 1:** Expected outcomes of resilience at the individual, team, and organizational levels.

**Level**	**Construct**	**Definition**	**Supporting authors**
Individual	Psychological health	Decreased prevalence of stress-related diseases such as Post-Traumatic Stress Disorder and Complicated Grief. Alternatively, resilience has also been associated with faster recovery from these diseases if they should occur.	McNally, 2003; Holland et al., 2009; Bonanno and Diminich, 2013
	Physical health	Decreased prevalence of physical disease following stress; increased pain tolerance; improved recovery from illness.	Rutter, 1998; Sturgeon and Zautra, 2013
	Sustained social ability	The ability to maintain effective relationships and demonstrate appropriate social skills in the face of stress.	Criss et al., 2015
	Sustained cognitive ability	The ability to collect, process, and act on information during or following periods of extreme stress.	Shia et al., 2015
Team	Maintenance of performance	Ability to maintain high levels of performance in spite of task challenges or difficulties.	Wilson et al., 2006
	Error avoidance	The prevention and/or minimization of errors.	Shawn Burke et al., 2005
	Desire to remain	Desire by team members to remain as part of the team.	Hackman and Wageman, 2005
Organizational	Maintenance of performance	Ability to maintain high levels of performance in spite of task challenges or difficulties.	Vogus and Sutcliffe, 2007
	Error avoidance	The prevention and/or minimization of errors.	Brown, 2004; Jeffcott et al., 2009
	Desire to remain	The extent to which an individual wishes to remain a member of the organization.	Kim and Aldrich, 2002; Majchrzak et al., 2007
	Sustained results	The ability to duplicate results each time a strategy is implemented.	Averett, 2001; Lissack and Letiche, 2002
	Longevity	Timespan indicative of the organization's success in its business environment in the past.	Linnenluecke and Griffiths, 2010

## Defining inputs of resilience

The inputs to resilience vary greatly depending on the level at which it is being considered. Table 2 summarizes the major inputs that enable resilience, again ordered by whether they occur at the individual, team, or organizational level. At the individual level, inputs to resilient behavior are most often considered to be individual traits. These traits serve to buffer individuals to the effects of a stressor and/or allow him or her to bounce back quickly. At the team level, inputs to resilience are not traits, rather they are factors that exist at the team level. However, they operate in a similar manner to individual inputs in that they can have a buffering effect on the team's experience of stress and/or equip them to cope with the stress. Finally, at the organizational level, input factors are similar to team-level inputs in that they exist at the organizational level and serve to set the stage for coping behaviors by the organization.

**Table 2:** Inputs that enable resilience.

**Level**	**Construct**	**Definition**	**Supporting authors**
Individual	Optimism	The tendency to anticipate a positive outcome, even in the face of adversity.	Riolli et al., 2002; Karademas, 2006
Individual	Personality	Refers to traits such as openness, agreeableness, emotional stability, and social competence.	Friborg et al., 2005
Individual	Goal orientation	A tendency to validate one's achievement ability in academic or performance settings.	VandeWalle et al., 2001
Individual	Coping flexibility	The ability to flexibly adjust coping strategies to face distinct stressors.	Lam and McBride-Chang, 2007; Galatzer-Levy et al., 2012
Individual	Coping	A dynamic situation-specific reaction to stress.	Lazarus, 1999; Eisenbarth, 2012
Individual	Self-esteem	A positive or negative attitude toward oneself.	Eisenbarth, 2012
Individual	Mental toughness	The ability to persevere through difficult circumstances and emerge without losing confidence.	Reivich et al., 2011
Individual	Directed attention	The ability to direct interpretations to a more flexible disposition.	Loprinzi et al., 2011; Sood et al., 2011
Individual	Cognitive restructuring	The modification of irrational thoughts.	Fava and Tomba, 2009
Individual	Sense of humor	Ability to find humor about life situations and about one's self.	Rutter, 1987; Bobek, 2002; Earvolino-Ramirez, 2007
Individual	Patience	The capacity to accept or tolerate delay, trouble, or suffering.	Connor, 2006
Individual	Faith	A belief in the doctrines of a religion.	Richardson, 2002; Ní Raghallaigh and Gilligan, 2010
Individual	Perseverance	Perceived ability to overcome adverse circumstances.	Floyd, 1996; Rolland and Walsh, 2006
Individual	Self-control	The capability to modulate and control impulses.	Moffitt et al., 2011
Individual	Hardiness	An openness to viewing change as a challenge.	King et al., 1998; Almedom, 2005
Individual	Grit	The passionate pursuit of long-term goals.	Duckworth et al., 2007
Team	Trust	The belief, confidence, or expectation that a fellow team member will be responsive and act in an ethically justifiable manner.	Meredith et al., 2011; Stephens et al., 2013
Team	Explicit communication	The transmission of ideas, knowledge, and thoughts to the receiving party between two or more team members via a verbal channel.	Entin and Serfaty, 1999; Vidal et al., 2009
Team	Implicit communication	The transmission of ideas, knowledge, and thoughts between two or more team members via a nonverbal channel.	Entin and Serfaty, 1999; Paton and Jackson, 2002
Team	Norms	A standard or pattern or behavior that has been established amongst team members.	Morgan et al., 2013
Team	Transactive memory	A combination of knowledge held by individual team members and the collective awareness of individual team member knowledge.	Ilgen et al., 2005
Team	Psychological safety	A perception that one can speak up without repercussion.	Carmeli and Gittell, 2009; Carmeli et al., 2009
Team	Stability of membership	The extent to which team members wish to remain as part of the team.	Kim and Aldrich, 2002; Majchrzak et al., 2007
Team	Assertiveness	The ability of a team member to communicate in a persuasive manner to other team members.	Wilson et al., 2005
Organizational	Preoccupied w/failure	Engagement in the analysis of possible vulnerabilities.	Vogus and Sutcliffe, 2007
Organizational	Agility	The ability to quickly and effectively cope with unexpected changes in the environment.	Lengnick-Hall and Beck, 2009; Fairbanks et al., 2014
Organizational	Monitoring	The ability to discern what is or is likely to become a threat in the near future.	Hollnagel et al., 2014
Organizational	Reluctance to simplify Interpretations	Tendency of an organization to question assumptions.	Vogus and Sutcliffe, 2007
Organizational	Sensitive to operations	A willingness to discuss the capabilities that facilitate safe performance.	Vogus and Sutcliffe, 2007
Organizational	Committed to resilience	The demonstration of effort to collectively learn from errors that have occurred.	Vogus and Sutcliffe, 2007
Organizational	Deference to expertise	The ability to migrate decisions to the person(s) with the greatest expertise for the issue at hand.	Vogus and Sutcliffe, 2007
Organizational	Adaptive capacity	A measure of dynamics of an organization that allows it to make decisions in both daily situations and crisis situations.	McManus et al., 2008; Lengnick-Hall et al., 2011
Organizational	Situation awareness	An understanding of the make-up of the organization and how its components relate to each other.	McManus et al., 2008

## Processes associated with resilience

Similar to input factors, the processes associated with resilience behavior vary greatly depending on the level being considered. At the individual level, resilient processes are most often conceived of as adaptive behaviors. At the team and organizational levels, resilient processes are more closely associated with collective behavior by team members. Table 3 summarizes our review of the literature regarding processes associated with resilience.

**Table 3:** Review of processes associated with resilience.

**Level**	**Construct**	**Definition**	**Supporting authors**
Individual	Stress management	A technique aimed at controlling an individual's stress level; particularly chronic stress levels.	Steinhardt and Dolbier, 2008; Loprinzi et al., 2011; Sood et al., 2011
Individual	Relaxation/Breathing	Techniques designed to reduce the physiological stress response through controlled breathing.	Deckro et al., 2002; Dziegielewski et al., 2004
Individual	Social support	A safe environment where individuals are encouraged to share their thoughts and feelings with others.	Karademas, 2006; Reivich et al., 2011
Individual	Imagery/mental stimulation	The use of all senses to rehearse an event scenario mentally.	Arnetz et al., 2009
Individual	Mindfulness	A mental state in which an individual focuses attention on the present moment, while acknowledging one's feelings, thoughts, and bodily sensations without judgement.	Shapiro et al., 1998
Team	Forceful backup	The questioning of a decision for which contrary evidence can be provided; the verbalization of conflicting information.	Lamb et al., 2014
Team	Planning	Formulation of a preconceived way to deal with hazards, crises, or potentially unexpected adverse event.	Crichton et al., 2009; Lentzos and Rose, 2009
Team	Leadership	The process of a superior influencing subordinates to accomplish team goals.	Lugg and Boyd, 1993; Wing, 2005; Stewart and O'Donnell, 2007
Team	Adaptability	A functional change in response to altered environmental and situational contingencies.	Pulakos et al., 2006; Carmeli et al., 2013; Alliger et al., 2015; Morgan et al., 2015; Wright and Masten, 2015
Team	Compensatory behavior	The ability to step in and provide back-up behavior for team members when they are unable to perform the task independently.	Van Der Haar et al., 2008
Team	Performance monitoring	Team's ability to monitor individual members' and the team's performance.	Wilson et al., 2005
Team	Shared decision making	Decisions are made jointly by team leaders and subordinates.	Stokols et al., 2008
Organizational	Anticipation	Knowing what to expect in terms of developments, threats, and opportunities that may occur in the near future.	Woods, 2006
Organizational	Information sharing	Transmission of data between a sender and receiver.	Paulus and Nijstad, 2003
Organizational	Simulating	Practice of the handling of unlikely events.	Vogus and Sutcliffe, 2007
Organizational	Management of keystone vulnerabilities	Management of organizational aspects are likely to mitigate negative impacts of a crisis.	McManus et al., 2008
Organizational	Information gathering	The process of collecting data and information pertinent to the task.	Kendra and Wachtendorf, 2003; Somers, 2009
Organizational	Layoff avoidance	Retainment of employees.	Gittell et al., 2006
Organizational	Financial reserves	Retainment of financial resources available during a crisis.	Gittell et al., 2006
Organizational	Broad resource networks	Ability to form relationships with others who may share fundamental resources.	Werner and Smith, 2001; Lengnick-Hall et al., 2011
Organizational	Diffused power	Reliance on self-organization for the creation of a holographic structure.	Lengnick-Hall et al., 2011
Organizational	Strategic HR management	Development of the requisite knowledge, skills, abilities, and other abilities (KSAOs).	Lengnick-Hall et al., 2011
Organizational	Enterprise systems	Large-scale packages that support organizational processes and information flows in complex organizations.	Ignatiadis and Nandhakumar, 2007
Organizational	Relational reserves	The maintenance of positive social relationships within the organization.	Gittell et al., 2006

## A comprehensive model of team resilience

**Figure 1:**
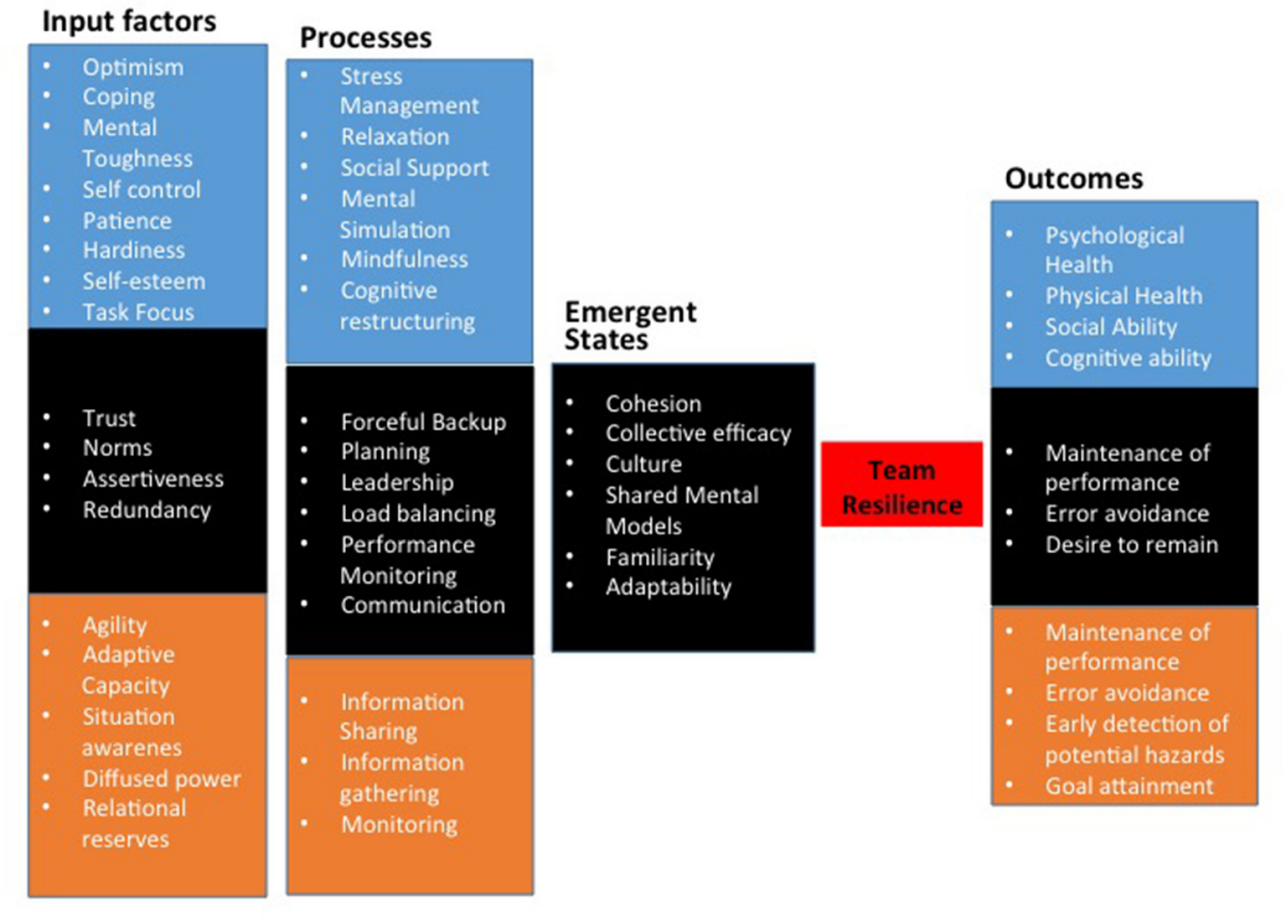
Summary of variables affecting resiliency.

Figure 1 displays a summary of the variables included in the tables above. As noted previously, we conceptualize team resilience as a second *order mediator*. That is, team resilience is best thought of as enabled by a combination of other team emergent states including cohesion, collective efficacy, culture, shared mental models, familiarity, and adaptability (see Table 4). Our conclusion is based on the notion that resilience is the result of these other states and it enables the team to achieve either positive or negative outcomes. It is this quality of resilience that is unique in that it can act as a buffer for negative outcomes and also as an enabler of positive ones.

**Table 4:** Team emergent states.

**Level**	**Construct**	**Definition**	**Supporting authors**
Team	Task adaptability	Ability of the team to shift their strategy to meet new or changing task demands.	Cannon-Bowers and Salas, 1998
Team	Cohesion	An engagement in and commitment to a group.	Schmidt et al., 2009; West et al., 2009; Weaver et al., 2011
Team	Collective efficacy	A group's shared belief in its capability to successfully complete a task or achieve a goal.	Morgan et al., 2013
Team	Culture	An established set of norms, rules, and behaviors that individuals within a team create for themselves.	Drinka, 1994; Morgan et al., 2013
Team	Shared mental models	A mental representation of a task, process, organization, or the team itself shared amongst team members.	Entin and Serfaty, 1999; Paton and Jackson, 2002
Team	Familiarity	Extent to which team members have personal knowledge of each other's strengths, weaknesses, preferences, styles, etc.	Smith-Jentsch et al., 2009
Team	Resilience	A dynamic process engaged in during the face of significant adversity, resulting in positive adaptation.	Luthar et al., 2000

**Figure 2:**
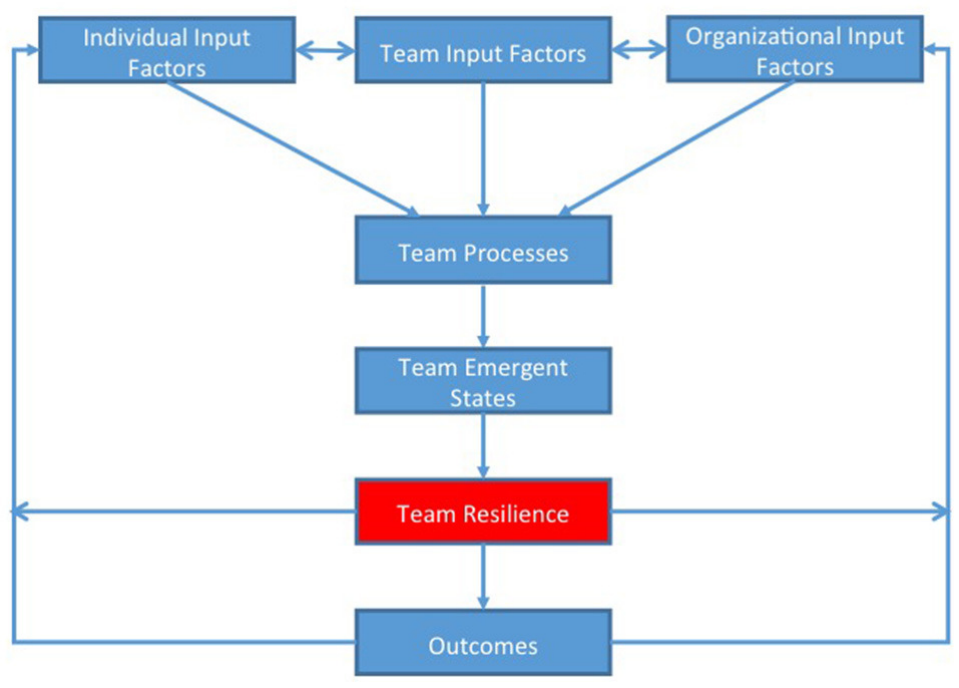
Visual depiction of model.

Inspection of the model in Figure 2 reflects what we have discussed above. According to this model, team resilience is a second order emergent state that is situated between other team emergent states (see Figure 1) and outcomes (see Figure 1). Team emergent states are the result of various team processes (see Figure 1) and those, in turn, are driven by input factors at the individual, team, and organizational level. We believe that this conceptualization is reflective of the complex, multi-level, dynamic relationship among variables at the team level.

## Research directions

The construct of emergent states allows researchers to propose hypotheses that better represent the dynamic, evolving nature of team processes and performance. However, given that this is a relatively new approach, empirical research to identify key emergent states is in its infancy. As this theoretical position is articulated, and as we develop new statistical tools to allow us to validate these models, we are learning more about the complex nature of team processes and the psychological states that result from team interactions.

In this paper, we have suggested a “second-order” emergent state of team resilience which might help us to understand how certain teams are able to cope with extreme stressors and to maintain their performance. More specifically, as an emergent state, this suggests that resilience may be the *result* of a number of team actions or processes, rather than a process in it of itself. Additionally, given the process vs. state debate in the literature, the nature of the construct of team resilience is certainly unclear. Thus, a new conceptualization of team resilience is warranted. As articulated throughout the present work, viewing team resilience as an emergent state may offer insight into the nature of team resilience that prior conceptualizations have failed to achieve. Ultimately, this is a hypothesis that will need to be validated using modeling approaches. However, it is important to articulate specific relationships that will be the foundation of these models. To that end, we propose the following first-order emergent states that we hypothesize will be related to the second-order emergent state of team resilience.

1. Collective Efficacy: Collective efficacy is typically defined as the team shared belief that it possesses the capability to achieve its goal. The relationship between collective efficacy and team performance has been demonstrated several times (see Gully et al., 2002, for a review). Collective efficacy is thought to work by influencing the amount of effort that team members are willing to invest and the degree of frustration they are willing to tolerate in pursuing team goals (Gully et al., 2002). These mechanisms are likely to be particularly important during times of high stress (Jex and Gudanowski, 1992). Therefore, we hypothesize that the emergence of collective efficacy will be positively related to team resilience. This position is supported by the results of Sharma and Sharma (2016) who included collective efficacy as a latent factor in their measurement model of team resilience. Similar support was reported by Morgan et al. (2013).
2. Team Cohesion: Similar to collective efficacy, team cohesion is an attitudinal state that is related to the degree to which team members value being in the team and their commitment to remaining in the team. Although, team cohesion is thought to also exert its influence through motivation, research has indicated that it is likely a different construct than collective efficacy (Paskevich et al., 1999). Specifically, team cohesion may influence performance through elements of mutual trust and the acceptance of, and adherence to, group norms (Carron et al., 2002). Adherence to group norms is an element that is thought to be a critical element in maintaining team performance under periods of high stress (Stevens et al., 2015). Cohesion is often included in theories of team resilience (Hind et al., 1996; Meredith et al., 2011). For example, Morgan et al. (2013) describe it as an element of collective efficacy. However, we might argue that it is better included in their construct of group identity. Nevertheless, it seems reasonable to suggest that cohesion is related to the emergence of resilience.
3. Shared Mental Models: Shared mental models have been defined as a collective representation of a task, process, organization, or team (Entin and Serfaty, 1999). Shared mental models have been linked to team performance under stress because they allow team members to coordinate their activities with the cognitive load of overt communication (Rouse et al., 1992). Several empirical studies have indicated the importance of shared mental models in allowing teams to maintain their performance when confronted with stress (e.g., Bolstad and Endsley, 1999; Stout et al., 1999; Mathieu et al., 2000). Interestingly, the emergence of shared mental models is rarely considered in theories of team resilience. However, the empirical data suggest that they may be a critical first-order emergent state.
4. Team Adaptability: Team adaptability refers to the ability of the team to recognize that a given strategy is not working and to adapt their strategy to meet the new demands (Cannon-Bowers and Salas, 1998). Team adaptability encompasses a number of behaviors and abilities that involve monitoring, problem-solving, and so forth. In fact, team adaptability is frequently used interchangeably with resilience in the lay literature. While similar, there are a few notable differences. First, we argue that adaptability is an emergent state that allows team members to perform in the short-term, whereas resilience allows them to grow and develop to facilitate performance in the longer term. Secondly, adaptive expertise has been defined as the ability to invent new procedures and make novel predictions based on extant knowledge (Hatano and Inagaki, 1986). Adaptation is considered to be evidenced when the individual responds successfully to changes in the task (Smith et al., 1997). However, resilience is typically demonstrated in response to adverse (rather than simply novel) events. It is a complex process comprised of processes whereby team members use their individual and collective resources to protect the group from stressors and positively respond when faced with adversity. As such, because resilience is independent of learning activities, it represents a greater repertoire of capabilities than adaptability alone. Finally, unlike the work on adaptability by Kozlowski and colleagues (e.g., Kozlowski et al., 1999, 2009) which places critical importance on the team leader, resilience also focuses on team development without emphasizing any particular team member. Instead, resilience work tends to place equal importance across all team members. In contrast, work by Kozlowski and colleagues places emphasis on how team *leaders* must build team capabilities. In particular, they note that planning and organizing, monitoring and acting are “executive *leadership* functions.” In the realm of resilience work, these tasks are also critical but equally distributed across team members. That said, there is no question that adaptability is a critical emergent state for the development of team resilience.

## Author contributions

All authors listed have made a substantial, direct and intellectual contribution to the work, and approved it for publication.

### Conflict of interest statement

The authors declare that the research was conducted in the absence of any commercial or financial relationships that could be construed as a potential conflict of interest.
